# Lactate-induced protein lactylation in cancer: functions, biomarkers and immunotherapy strategies

**DOI:** 10.3389/fimmu.2024.1513047

**Published:** 2025-01-10

**Authors:** Wenjuan Wang, Hong Wang, Qi Wang, Xiaojing Yu, Liangliang Ouyang

**Affiliations:** ^1^ Department of Medical Laboratory, Affiliated Hospital of Jiujiang University, Jiujiang, Jiangxi, China; ^2^ Co-Innovation Center for Sustainable Forestry in Southern China, College of Life Sciences, Nanjing Forestry University, Nanjing, China

**Keywords:** lactate, lactylation, post-translational modification, proteins interacting with lactate, cancer

## Abstract

Lactate, long viewed as a byproduct of glycolysis and metabolic waste. Initially identified within the context of yogurt fermentation, lactate’s role extends beyond culinary applications to its significance in biochemical processes. Contemporary research reveals that lactate functions not merely as the terminal product of glycolysis but also as a nexus for initiating physiological and pathological responses within the body. Lysine lactylation (Kla), a novel post-translational modification (PTM) of proteins, has emerged as a pivotal mechanism by which lactate exerts its regulatory influence. This epigenetic modification has the potential to alter gene expression patterns, thereby impacting physiological and pathological processes. Increasing evidence indicates a correlation between lactylation and adverse prognosis in various malignancies. Consequently, this review article aims to encapsulate the proteins that interact with lactate, elucidate the role of lactylation in tumorigenesis and progression, and explore the potential therapeutic targets afforded by the modulation of lactylation. The objective of this review is to clarify the oncogenic significance of lactylation and to provide a strategic framework for future research directions in this burgeoning field.

## 1 Introduction

Energy serves as the linchpin for sustaining life processes, with glucose emerged as the primary energy currency powering the vitality of all mammals. The generation of energy from glucose is chiefly orchestrated through two interconnected metabolic pathways: glycolysis and oxidative phosphorylation. Conventionally, lactate was perceived as a metabolic byproduct of glycolysis that accumulated under hypoxic conditions (1). However, in the 1920s, Otto Warburg seminally revealed a distinct anomaly in the energy metabolism of cancer cells: tumor tissues exhibit an anomalously high consumption of glucose compared to their adjacent normal counterparts. This observation underscored the paradoxical preference of tumor cells for aerobic glycolysis, even in the presence of sufficient oxygen, as a means to swiftly acquire energy. This distinct metabolic phenotype, characterized by the preferential conversion of glucose to lactate, regardless of oxygen availability, is now known as the Warburg effect (2–4). As our comprehension of cellular metabolism has evolved, it has become apparent that the phenomenon of aerobic glycolysis is not confined to cancer cells alone. It is also observed in conditions such as sepsis (5–7), cardiovascular diseases (8–10), inflammatory diseases (11, 12), and autoimmune diseases (13–16). This revelation underscores the pivotal role that lactate plays in the pathophysiological processes underlying these diverse clinical conditions.

In 2019, lactate garnered public attention with a novel “posture”, following the discovery of a novel PTM of proteins by Professor Yingming Zhao’s team, termed “lactylation” (17). This breakthrough was achieved through the application of high-resolution liquid chromatography-tandem mass spectrometry (LC-MS/MS) analysis, which detected the presence of histone Kla for the first time. The analysis revealed the presence of 26 histone Kla sites in human HeLa cells and 16 sites in mouse bone marrow-derived macrophages (BMDM), encompassing histones H3, H4, H2A, and H2B (17). The enzymatic reaction responsible for Kla is mediated by the presence of the lysine acetyltransferase (KAT) enzyme P300 and requires lactyl-CoA. L-lactyl-CoA, the activated form of L-lactate, emerges as a crucial substrate for this modification process. This discovery adds a new layer of complexity to our understanding of protein modification and the roles of lactate in cellular metabolism.

Recent research has unequivocally established the role of lactate in facilitating cancer progression (18). The advent of lactylation has introduced a novel dimension to our understanding of lactate’s functions within tumor cells. To delve more deeply into the implications of lactylation on cancer biology, this review article surveys the current literature, seeking to elucidate the role of lactylation in tumor cells, investigate the potential for targeted modulation of lactate and lactylation in cancer therapy, and to offer insights that could inform the development of novel combined targeted and immunotherapeutic strategies against cancer.

## 2 Lactate metabolism

**Figure 1:**
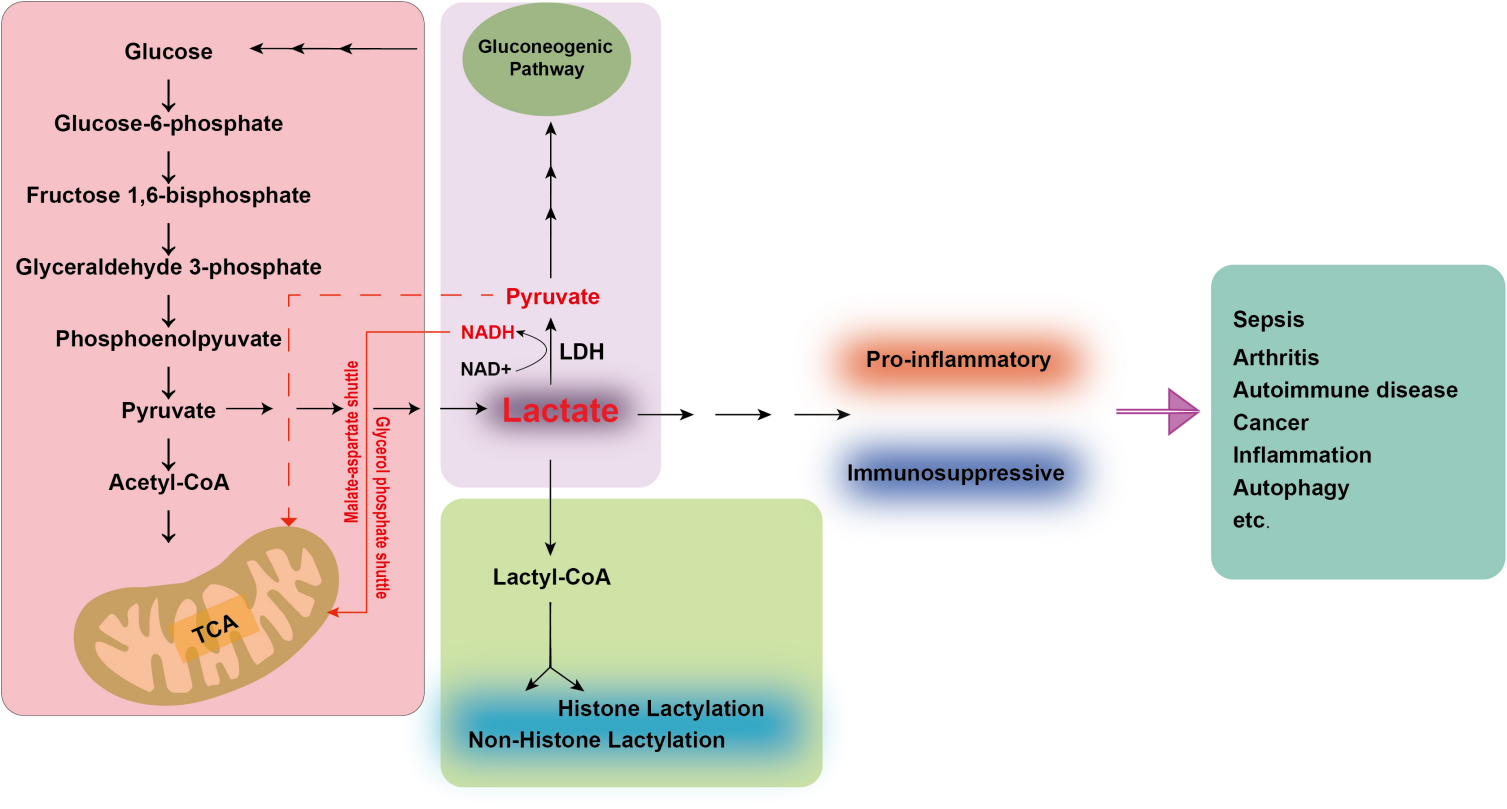
Lactate metabolism and regulation of physiological and pathological functions.

Lactate is conventionally understood as a metabolite generated through the glycolytic pathway under anaerobic conditions. Despite its historical reputation as a waste product, lactate indeed fulfills a vital role in physiological metabolism. Nature encompasses two stereoisomers of lactate: L-lactate and D-lactate. D-lactate is predominantly derived from dietary sources, whereas L-lactate, a common byproduct of human metabolism, is not only produced within the body but is also readily absorbed and utilized. Glucose has emerged as the primary energy currency of the cell. Glucose is metabolized to yield two molecules of pyruvate primarily through the glycolytic pathway. Under oxygen-deprived conditions, lactate dehydrogenase (LDH) catalyzes a critical reaction, reducing pyruvate to L-lactate using Nicotinamide adenine dinucleotide (NADH) as a cofactor. This process yields a net gain of two ATP molecules and two lactate molecules per glucose molecule metabolized, while consuming no oxygen (19). In contrast, under aerobic conditions, the NADH and pyruvate produced during glycolysis are shuttled into the mitochondria, where they participate in oxidative phosphorylation, generating significantly more energy (over a dozen times more) than under anaerobic conditions. Furthermore, lactate is transported via the bloodstream to the liver, where it is converted back to glucose through a process known as the lactate cycle or Cori cycle. This cycle represents a recycling mechanism that converts glucose into lactate and vice versa, thereby providing the body with additional energy sources. Lactate, however, also serves as an energy substrate, meeting the energy demands of neurons when blood glucose levels are insufficient (20). It provides signaling cues that regulate neuronal function and serves as a crucial carbon source for the tricarboxylic acid (TCA) cycle (21). Simultaneously, lactate functions as a redox signaling molecule, mediating communication between cells and tissues and playing a significant role in cellular metabolism (22) (**Figure 1**).

During glycolysis, glucose is metabolized to pyruvate. In normoxic conditions, pyruvate enters the mitochondria and is completely oxidized to CO_2_ and H_2_O via the TCA cycle. In hypoxia or intense glycolysis, pyruvate is reduced to lactate by LDH Lactate is subsequently converted to Lactyl-CoA, facilitating lactylation of histones and non-histone proteins, potentially influencing their function and stability. Lactate also serves as a substrate for gluconogenesis, which enables the cellular resynthesis of glucose. Concurrently, LDH can convert lactate into pyruvate, serving as a primary fuel for the TCA cycle. The extra NADH molecules generated can be shuttled into the mitochondria through the malate-aspartate and glycerol phosphate shuttles. This conversion serves as a metabolic adaptation to preserve energy and reduce the build-up of toxic byproducts. In disease states, the acidic environment sustained by lactate can contribute to immunosuppressive or pro-inflammatory conditions, thereby exacerbating the progression of diseases such as sepsis, arthritis, autoimmune disorders, and cancer. TCA, tricarboxylic acid; LDH, lactate dehydrogenase; NADH, Nicotinamide adenine dinucleotide.

Clinically, lactate has been employed as a biomarker to monitor the prognosis of critically ill patients. Recent research has established that lactate serves as a critical signaling molecule in immune regulation (23–26). The accumulation of lactate within the microenvironment markedly influences the activity and function of immune cells, including tissue-resident and infiltrating immune cells. Elevated lactate levels within this microenvironment can lead immune cells into an immunosuppressive state or augment inflammatory signals in inflamed tissues, aiding in evading immune surveillance (3, 24, 27–29). In a mouse model of arthritis, lactate can upregulate the expression of the lactate transporter SLC5A12 on CD4 T cells, which in turn enhances IL-17 production and fatty acid synthesis through the nuclear PKM2/STAT3 signaling pathway (30). Concurrently, in rheumatoid arthritis (RA), CD8+ T cell subsets overexpress lactate dehydrogenase A (LDHA), and targeting LDHA activity to remodel glucose and glutamine metabolism in RA CD8+ T cells can mitigate the inflammatory and destructive role of these cells in the progression of autoimmune diseases (31). Furthermore, lactate curtails the production of inflammatory cytokines and mast cell degranulation *in vitro*, thereby limiting the mast cell-mediated inflammatory response (32). Simultaneously, lactate curbs TLR-mediated activation of monocyte-macrophages, retards the phosphorylation of protein kinase B (AKT), and inhibits the degradation of IκBα. It also suppresses the secretion of cytokines TNF-α and IL-23, as well as chemokines CCL2 and CCL7 (33). In septic acute kidney injury (AKI), lactate can activate the PD-1/PD-L1 pathway, leading to lymphocyte apoptosis and immunosuppression (34). As increasing focus is directed toward tumor immunity, it has been observed that immunosuppression plays a pivotal role in tumor proliferation and invasion. The tumor microenvironment (TME) is hypoxic and rich in lactate. Tumor-derived lactate differentiates macrophages into an M2 phenotype, which can facilitate tumor initiation (29, 35–37) and progression or dysfunction other immune cells (28, 38–41), thereby inducing immunosuppression and aiding tumor immune evasion, thereby supporting tumor growth and survival, ultimately resulting in a poor patient prognosis (42–45).

## 3 Lactate-interacting proteins

**Figure 2:**
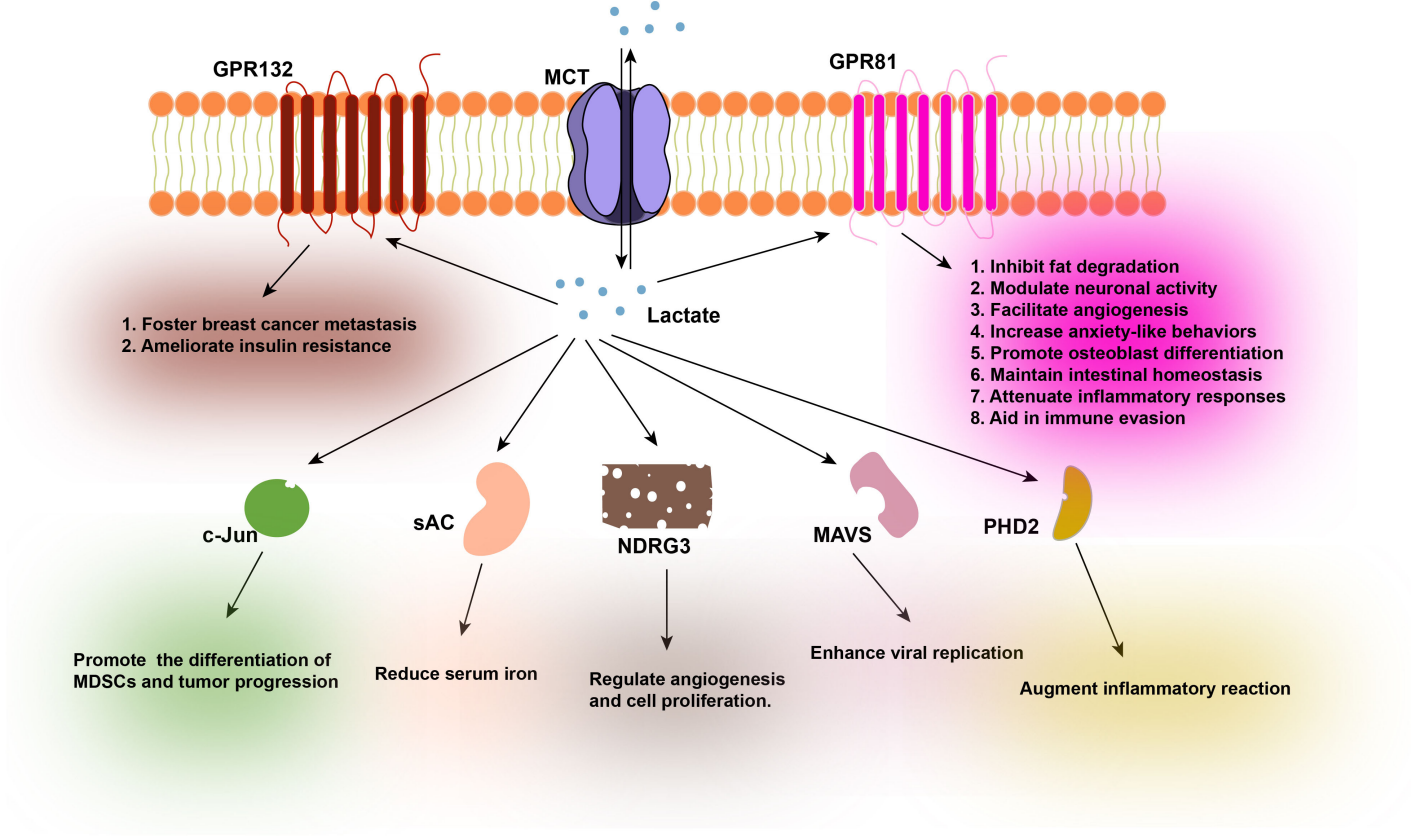
Functions of various proteins after their interaction with lactate.

As a pleiotropic signaling molecule, lactate is capable of orchestrating a diverse array of biological processes, encompassing immune inflammatory responses (46), metabolic pathways (22), angiogenesis (47, 48), and intercellular coordination within the TME (49, 50). The movement of lactate across cellular membranes is primarily facilitated by the Monocarboxylate transporter (MCT) proteins, which are integral to the cell membrane (51). The activity of these transporters is exquisitely tuned to the lactate concentration gradient across the cell membrane. In environments where the extracellular lactate concentration is high, such as in the TME or inflamed tissue, the uptake of lactate into cells prevails. Conversely, when cellular levels of lactate are elevated, the export of lactate becomes predominant. A wealth of research has underscored the pivotal role of lactate in orchestrating a multitude of biological and pathological processes. Furthermore, studies have demonstrated that lactate can directly interact with cellular proteins, thereby mediating its biological impact (**Figure 2**).

Lactate originates from two primary sources: endogenous production within cells and exogenous intake. The transportation of lactate across the cell membrane is predominantly facilitated by Monocarboxylate Transporters (MCTs). Lactate functions as a signaling molecule and interacts with a variety of protein ligands, including GPR132, GPR81, c-Jun, sAC, NDRG3, MAVS, and PHD2, among others. The interaction of GPR132 with lactate fosters breast cancer metastasis and ameliorates insulin resistance by inducing macrophage phenotypic shifts. The interaction of GPR81 with lactate has been demonstrated to inhibit fat degradation, modulate neuronal activity, facilitate angiogenesis, increase anxiety-like behaviors, promote osteoblast differentiation, maintain intestinal homeostasis, attenuate inflammatory responses, and aid in immune evasion. The interaction of c-Jun with lactate promotes the differentiation of Myeloid Derived Suppressor Cells (MDSCs), contributing to tumor progression. The interaction of sAC with lactate can lead to reduced serum iron levels, which may exacerbate iron-deficiency anemia. The interaction of NDRG3 with lactate plays a regulatory role in angiogenesis and cell proliferation. The interaction of MAVS with lactate compromises the innate antiviral response, thereby enhancing viral replication. Lastly, the interaction of PHD2 with lactate augments inflammatory reactions in adipose tissue macrophages. GPR132, G-protein coupled receptor 132; MCTs, Monocarboxylate Transporters; GPR81, G-protein coupled receptor 81; c-Jun, Jun proto-oncogene; sAC, Soluble Adenylyl Cyclase; NDRG3, N-myc downstream regulated gene 3; MAVS, Mitochondrial Antiviral Signaling Protein; PHD2, Prolyl Hydroxylase Domain 2.

### 3.1 GPR132

G-protein coupled receptor 132 (GPR132) is expressed across various tissues and immune cell populations, where it plays a pivotal role in the regulation of a spectrum of cellular biological processes, including the cell cycle, cell proliferation, and immune responses (52). Extensive research has underscored the significance of the lactate-GPR132 axis in driving the metastatic potential of breast cancer, primarily by facilitating tumor-macrophage interactions. lactate has been shown to bind to macrophage GPR132, thereby promoting the polarization of macrophages towards the M2 phenotype, which in turn enhances the adhesion, migration, and invasive capabilities of breast cancer cells (53). Additionally, lactate can interact with GPR132 receptors on the surface of adipose tissue macrophages (ATMs) in mice, inhibiting the pro-inflammatory M1 polarization of ATMs through the activation of the GPR132-PKA-AMPKα1 signaling pathway. Consequently, this mechanism can ameliorate insulin resistance in mice fed a high-fat diet (HFD) (54).

### 3.2 GPR81

G-protein coupled receptor 81 (GPR81), also known as Hydroxycarboxylic acid receptor 1 (HCA1), is a member of the G protein-coupled receptor (GPCR) family that is expressed on the cell membrane. Early studies identified L-lactate as a natural ligand for GPR81 in adipocytes, which interacts directly with the GPR81/Gi pathway to downregulate cAMP levels and promote lipid accumulation within these cells (55, 56). As research progresses, the roles of lactate and GPR81 are increasingly recognized in a wide range of physiological and pathological processes.

Recent findings have demonstrated lactate binding to GPR81 in the brain and retina, suggesting its potential as a signaling molecule for neuroprotection, angiogenesis, and neuronal activity modulation (57–61). Furthermore, gut microbiota-derived lactate has been associated with anxiety-like behaviors in rodents through GPR81-mediated lipid metabolism pathways (62). Lactate has also been shown to activate the GPR81-Gβγ-PLC-PKC-Akt signaling axis, which regulates osteoblast differentiation in response to parathyroid hormone (PTH) treatment (63). The interaction between lactate and GPR81 can modulate the inflammatory response during labor, with high levels of lactate during labor potentially downregulating key proinflammatory genes in the uterus (64). The lactate-GPR81 axis has implications in innate immune suppression (65–67) and has been shown to negatively regulate TLR-induced NLRP3 inflammasome activation and IL-1β production, thereby reducing liver and pancreatic injury in TLR and inflammasome-mediated inflammation (65). Lactate binding to GPR81 inhibits the activation of YAP and NF-κB through signal transduction, dampening the pro-inflammatory response of macrophages to LPS stimulation (66). Additionally, lactate has been found to inhibit the proinflammatory response and metabolic reprogramming of macrophages in a GPR81-independent manner (67). The binding of lactate to GPR81 is critical for maintaining intestinal homeostasis, as lactate derived from lactic-acid-producing bacteria (LAB) symbionts can promote intestinal stem cell (ISC)-mediated epithelial development, thereby balancing intestinal homeostasis (68). Physiologically relevant doses of lactate have been shown to rescue the oscillatory shear stress (OSS)-induced decrease in GPR81 expression, reduce oxidative stress, and inhibit OSS-induced endothelial inflammation by activating Kruppel-like factor 2 (KLF2) expression, which may prevent the development of atherosclerosis (69). In the TME, the interplay between GPR81 and high levels of lactate is crucial for tumor immune evasion, proliferation, and metastasis. Studies have indicated that GPR81 is highly expressed in various cancer cell lines, including colon, breast, lung, hepatocellular, salivary gland, cervical, and pancreatic cancers, enhancing the metastatic and invasive abilities and environmental adaptability of cancer cells. This underscores the significant role that the lactate-GPR81 axis plays in the occurrence and development of tumors (70–74).

### 3.3 c-Jun

Jun proto-oncogene (c-Jun), a critical transcription factor indispensable for the expression of COX2, has been identified to interact with lactate. This interaction serves to obstruct the binding of c-Jun to FBW7, a protein that facilitates ubiquitination and subsequent degradation of c-Jun. By associating with lactate, c-Jun is shielded from FBW7-mediated ubiquitination and degradation, thereby stabilizing c-Jun and enhancing its transcriptional activity. The subsequent activation of c-Jun signaling leads to an upregulation of COX2 expression, which in turn regulates the differentiation of granulocytic MDSCs. This process ultimately promotes tumor progression (75).

### 3.4 sAC

The soluble adenylyl cyclase (sAC) serves as the enzymatic executor tasked with catalyzing the conversion of ATP to the pivotal secondary messenger, cyclic AMP (cAMP) (76, 77). Studies within the domain of iron homeostasis have revealed that individuals experiencing hyperlactatemia frequently present with symptoms akin to those of anemia (78). Within normal hepatocytes, lactate binds to sAC, thereby stimulating its enzymatic function, which in turn elevates cAMP levels. This increase in cAMP facilitates the upregulation of hepcidin transcription through the cAMP/PKA/Smad1/5/8 signaling cascade. Consequently, this mechanism inhibits the absorption of dietary iron from the gastrointestinal tract and the export of iron from macrophages, leading to a reduction in serum iron levels and potentially exacerbating the symptoms of iron deficiency anemia (78).

### 3.5 NDRG3

Under normal growth conditions, N-myc downstream regulated gene 3 (NDRG3) undergoes hydroxylation by PHD2 and ubiquitinated for degradation by VHL, a targeted element of the E3 ubiquitin ligase complex. However, under hypoxic conditions, it has been observed that NDRG3 forms a complex with lactate, which serves to protect NDRG3 from VHL-mediated degradation. Furthermore, NDRG3 has been demonstrated to bind to c-Raf, thereby facilitating the phosphorylation and activation of the c-Raf-ERK signaling pathway. This activation is instrumental in promoting angiogenesis and cell growth (43).

### 3.6 MAVS

Mitochondrial antiviral-signaling protein (MAVS) functions as a direct sensor for lactate, mediating the integration of energy metabolism and innate immunity. Upon pathogen entry, the genetic material of the pathogen serves as a key stimulus for the activation of the innate immune response. Research has demonstrated that MAVS is downstream of retinoid acid-induced gene I (RIG-I) and serves as a critical bridge between antiviral innate immunity and glycolysis within the RIG-I-like receptor (RLR) signaling cascade. The direct interaction between hexokinase 2 (HK2) and MAVS, as well as that between lactate and MAVS, plays a pivotal role in the antiviral immune response. During viral infection, lactate can bind directly to the transmembrane domain of MAVS, inhibiting its polymerization and thereby facilitating the hijacking of RIG-I-MAVS by HK2-MAVS, which disrupts the normal glycolytic process and inhibits the downstream signaling of type I interferon (79, 80). Recent investigations have revealed that Senecavirus A (SVA) can enhance viral replication by stimulating lactate production, and it also reduces the interaction between MAVS and RIG-I, thereby attenuating the antiviral response (81).

### 3.7 PHD2

Prolyl hydroxylase domain-containing protein 2 (PHD2) is an oxygen sensor protein that resides within cells, serving as a molecular sentinel for alterations in oxygen partial pressure, with its enzymatic activity being exquisitely sensitive to intracellular oxygen levels. In normoxic conditions, hypoxia-inducible factor α (HIFα) serves as a classical regulatory substrate for prolyl hydroxylases (PHDs), undergoing hydroxylation by these enzymes. Subsequently, HIFα is targeted for degradation through the protein ubiquitination pathway, which is mediated by E3 ubiquitin ligases. Conversely, under hypoxic conditions, the activity of PHDs is repressed, leading to the persistent activation of HIFα (82–85). In adipose tissue, adipocyte-derived lactate emerges as a key player. At physiological concentrations, lactate competitively replaces α-ketoglutarate, directly binding to the catalytic domain of PHD2. This interaction enhances the activation of ATMs, Consequently, this stabilizes HIF-1α and amplifies the inflammatory response of adipose tissue macrophages (86).

## 4 Lactylation is a dynamic process

As a novel PTM of histones, lactylation represents an intricate and dynamic process deeply entrenched in cellular metabolism. This modification is predominantly governed by cellular metabolism and enzymes associated with lactylation.

### 4.1 Cellular metabolism

It has been observed that intracellular lactate modulates the levels of histone Kla (H3K18la and H4K5la) in a dose-dependent fashion, with both exogenous and endogenous lactate capable of enhancing Kla. Manipulations that curtail lactate generation or impede glycolysis tend to reduce the incidence of Kla, conversely, those that augment lactate production or stimulate glycolysis promote such modification.

### 4.2 Writers

The lactylation “writers” are key enzymes tasked with catalyzing the lactylation process. They are pivotal in the addition of lactyl groups to specific lysine residues, which in turn can perturb chromatin structure and regulate gene expression. These enzymes are central to various biological processes involving lactylation.

#### 4.2.1 P300

Recent scholarly inquiries have elucidated that lactate-driven lactylation is a responsive and altering enzymatic reaction. P300, an acetyltransferase, is responsible for transmitting the acetyl moiety from acetyl-CoA to the lysine residues of histones, thereby causing acetylation. Moreover, P300 emerges as a pivotal enzyme in the cascade of events leading to lactylation, as it can catalyze the transfer of the lactyl group from lactyl-CoA to the lysine residues of histones, resulting in the lactylation of histones. Elevated expression of P300 has been shown to markedly enhance the extent of lactylation (17). In contrast, knocking down P300 expression in macrophages caused a significant decrease in the levels of Kla (87), and inhibiting the expression of P300/CBP reduced the Kla levels of HMGB-1 (5).

#### 4.2.2 KAT8

KAT8 is a lactyltransferase enzyme that plays a critical role in the PTM of proteins, specifically in the installation of lactyl groups on protein substrates. This modification is implicated in various biological processes, including tumorigenesis. In the context of colorectal cancer (CRC), KAT8 has been identified as a key regulator that promotes cancer progression by facilitating the lactylation of eukaryotic translation elongation factor eEF1A2 at lysine 408. This lactylation event enhances protein translation efficiency, thereby contributing to the tumorigenic potential of CRC. Importantly, the manipulation of KAT8 expression, through knockdown techniques or the use of specific inhibitors, has been shown to significantly inhibit tumor growth in experimental models (88). Furthermore, research has also uncovered a role for KAT8 in mediating the lactylation of latency-transforming growth factor beta-binding protein 1 (LTBP1). This modification is involved in the induction of collagen synthesis in fibroblasts, which is purported to contribute to skin rejuvenation. The interplay between KAT8-regulated lactylation and tissue homeostasis underscores the multifaceted role of this enzyme in biological systems and highlights its potential as a therapeutic target in various pathological conditions (89).

#### 4.2.3 AARS1

Recent investigations have elucidated that Alanyl-tRNA Synthetase 1 (AARS1) possesses lactyltransferase activity both *in vitro* and *in vivo*, capable of catalyzing protein lactylation using lactate and ATP as substrates. In the context of gastric cancer cells, AARS1 senses an increase in intracellular lactate levels and translocates to the nucleus, where it directly lactylates the YAP-TEAD complex. This modification activates downstream gene expression, thereby promoting tumor cell proliferation (90). Furthermore, AARS1 catalyzes the lactylation of specific lysine residues within the DNA-binding domain of p53, which in turn diminishes the tumor suppressor function of p53 (91).

#### 4.2.4 AARS2

Alanyl-tRNA Synthetase 2 (AARS2) is a protein lysine lactyltransferase whose expression level is regulated by hypoxia-induced PHD2. Prior research has demonstrated that hypoxic conditions result in the accumulation of AARS2, which catalyzes the lactylation of PDHA1 and carnitine palmitoyltransferase 2 (CPT2) within the pyruvate dehydrogenase complex. This modification leads to the inactivation of these enzymes, subsequently inhibiting oxidative phosphorylation (OXPHOS) (92). Additionally, AARS2 is capable of transferring the lactylation modification to the cGAS protein, thereby inhibiting its activity and compromising the function of innate immune surveillance (93).

The aforementioned studies suggest that multiple “writers” are capable of catalyzing the lactylation of proteins. However, within the continually evolving research on lactylation writers, it is becoming apparent that the specific enzymes and their mechanisms of action can exhibit cell type and organismal dependencies. The identification and characterization of these writers are crucial for elucidating the role of lactylation in cellular biology and disease pathogenesis.

### 4.3 Erasers

The lactylation “erasers” have the capacity to remove lactyl groups that have been imparted to lysine residues on proteins, thereby reversing lactylation modifications. This process can lead to alterations in chromatin structure and gene expression levels, potentially activating or silencing genes.

Histone deacetylases (HDACs), encompassing HDAC1-11 and SIRT1-7, form a family of enzymes with dual functionality. HDACs are not only capable of histone deacetylation but also exhibit delactylation properties (94). Specifically, HDAC1-3 have been found to significantly downregulate the expression of H3K18la and H4K5la, whereas SIRT1-3 exhibit only a modest reduction (94).

Currently, the field of research on lactylation “erasers” is in its infancy, yet initial studies are providing insights into the function and mechanism of these enzymes. This research is of profound importance for a comprehensive understanding of the role of lactylation in cellular biology, development, and the pathogenesis and therapy of diseases. By harnessing the ability to modulate these erasers, it is anticipated that novel therapeutic strategies can be devised to regulate lactylation-associated diseases, including cancer, in the foreseeable future.

Cell metabolism is pivotal in orchestrating the fate and function of cellular processes. While glycolysis has been extensively investigated within the context of various diseases’ pathophysiology, the emergence of lactylation has introduced novel insights into the metabolic repercussions of glycolytic activity. Lactylation exerts its influence on the physiological and pathological trajectories of diseases by employing mechanisms such as epigenetic regulation. At the gene promoter level, an increase in the prevalence of Kla facilitates the unwinding of the DNA double helix. This unwinding event creates an environment conducive to the binding of pertinent transcription factors and gene promoters. Consequently, this interaction enhances the transcription and translation of the genes in question, culminating in specific cellular effects.

## 5 Function of lactylation in cancers

**Figure 3:**
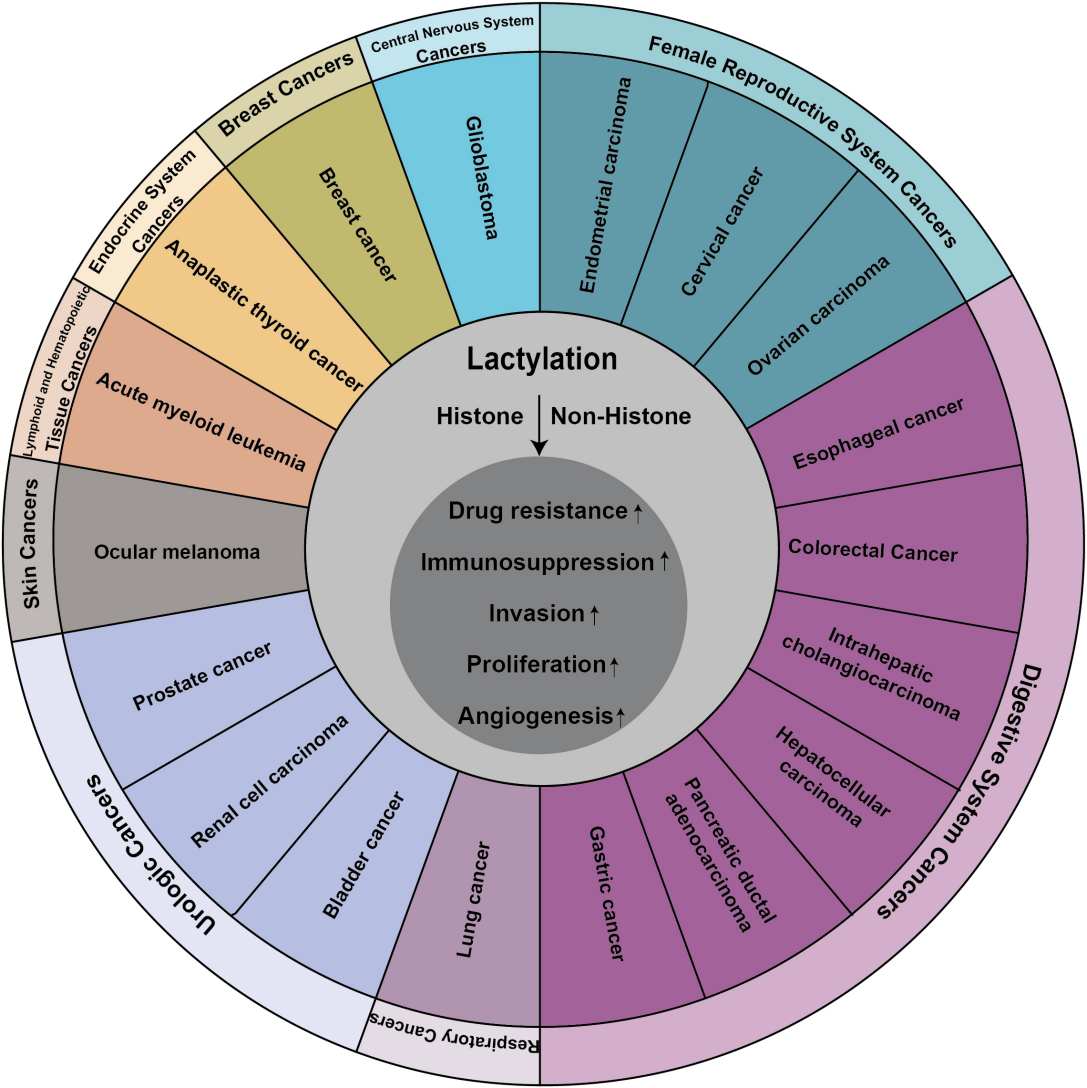
Lactylation contributes to the progression of many cancers.

Lactate, once considered a metabolic byproduct, has been rebranded as a crucial player in cellular metabolism following a seminal study that uncovered its role in histone lactylation (17). This posttranslational modification alters the perception of lactate, transitioning it from the proverbial “ugly duckling” to a “white swan”, thereby heralding new avenues for investigating the non-metabolic functions of lactate in both physiological and pathological contexts. Initially identified in the context of inflammation and immunity research, lactylation has since been implicated in a variety of diseases and pathological processes, garnering increasing attention from the scientific community (**Figure 3**).

The metabolic reprogramming of tumor cells is a pivotal process that facilitates the initiation and progression of cancer. Cancer cells strategically alter their metabolic pathways to independently satisfy heightened demands for bioenergy and biosynthesis, which not only fosters their rapid growth and proliferation but also mitigates the detrimental effects of oxidative stress (95). The accumulation of lactate within the TME has been identified as a source of energy that promotes tumor proliferation and invasion. The Warburg effect, a defining characteristic of tumor cells, underscores the inevitability of increased protein lactylation within tumor tissues, reflecting this metabolic adaptation’s integral role in cancer biology (25, 96–102).

### 5.1 Digestive system cancers

#### 5.1.1 Esophageal cancer

Esophageal cancer (EC) is a lethal gastrointestinal malignancy. Serine hydroxymethyltransferase 2 (SHMT2) constitutes one of the most prominently expressed metabolic enzymes in human carcinomas and is markedly elevated in EC tissues and cells. Hypoxia is shown to augment the lactylation of SHMT2, which stabilizes the enzyme and upregulates MTHFD1L expression, thereby expediting the aggressive progression of EC cells (103). Concurrently, hypoxic conditions have been demonstrated to foster an increase in histone H3K9la and potentiate the transcription of LAMC2, promoting the proliferation of esophageal squamous cell carcinoma (ESCC) (104). The upregulation of Axin1 has been observed to impede tumor growth and is implicated in the suppression of glycolysis. Hypoxia-induced lactylation at Axin1’s lysine 147 residue promotes its ubiquitination and subsequent protein degradation, thereby facilitating glycolysis and maintaining the stemness of esophageal cancer cells (105).

#### 5.1.2 Colorectal cancer

CRC ranks as the third most frequently diagnosed malignancy in both males and females (106), yet approximately 60-70% of patients exhibiting clinical symptoms have CRC at an advanced stage (107). Research has indicated that β-catenin is markedly upregulated in CRC tissues and cells, and hypoxia-induced glycolysis markedly augments the lactate levels controlled by β-catenin in CRC cells. The emergence of lactylation stabilizes and enhances the expression of β-catenin, thereby exacerbating CRC progression via the Wnt signaling pathway (108). Intestinal microbiota can contribute to carcinogenesis by epigenetically interacting with the host genome. During Salmonella typhimurium SL1344 infection, normal cells produce LINC00152, a long non-coding RNA (lncRNA). Elevated histone lactate levels in clinical CRC samples result in the overexpression of LINC00152, which promotes the metastasis and invasion of CRC cells (109). Diapause, characterized by a pause or reduction in cancer cell proliferation under stress conditions, is observed in SMC-related diapause CRC cells. These cells exhibit increased expression of key enzymes involved in glycolysis, leading to heightened lactate production and elevated levels of histone lactylation at H4K8, H4K12, and H3K14. This, in turn, modulates ABC transporter expression and enhances chemosensitivity in CRC diapause cells by inhibiting drug efflux (110, 111). Increased histone lactylation has been detected in bevacizumab-resistant CRC patients. H3K18 lactylation activates RUBCNL/Pacer transcription, promoting autophagosome maturation through BECN1 (beclin 1) interaction, which recruits and activates the class III phosphatidylinositol 3-kinase complex, triggering rapid proliferation and drug resistance in CRC cells (112). Recent studies have shown that tumor-derived lactate enhances H3K18 lactylation, inhibiting RARγ gene transcription in macrophages and thereby increasing interleukin-6 (IL-6) levels within the TME, activating the TRAF6-IL-6-STAT3 signaling pathway, which promotes tumor-promoting functions in macrophages (113). GPR37 expression is significantly elevated in CRC liver metastasis (CRLM) specimens and is associated with poor prognosis (114). GPR37 can activate the Hippo pathway, upregulate LDHA expression and glycolytic levels, and result in increased H3K18la abundance, leading to the upregulation of CXCL1 and CXCL5, ultimately contributing to colorectal cancer liver metastasis (114). KAT8 is the enzyme responsible for the assembly of Kla on numerous protein substrates involved in diverse biological processes. In CRC, KAT8 can induce the lactylation of eEF1A2K408, leading to enhanced translational elongation and protein synthesis, thereby promoting tumorigenesis (88). Increased expression of methyltransferase-like 3 (METTL3) in tumor-infiltrating myeloid cells (TIM) is associated with poor prognosis in colon cancer patients. It has been found that the accumulated lactate in the TME induces H3K18 lactylation-mediated upregulation of METTL3 expression in TIM cells, activating the JAK1-STAT3 signaling pathway in TIM. Surprisingly, METTL3 contains two lactylation sites in its zinc finger domain, at K281 and K345. METTL3 lactylation-mediated RNA m6A modification plays a crucial role in promoting the immunosuppressive ability of TIM, which, in turn, fosters the invasion and growth of cancer cells (115).

#### 5.1.3 Intrahepatic cholangiocarcinoma

Intrahepatic cholangiocarcinoma (ICC) represents the second most prevalent primary hepatic neoplasm and continues to be a lethal malignancy in the majority of patients (116). Nucleolin (NCL), a key RNA-binding protein predominantly located within the nucleolus, plays a significant role in gene regulation. The acetyltransferase P300 specifically catalyzes the lactylation of NCL at lysine 477, a PTM that facilitates its interaction with the primary transcript of the MAP kinase-activating death domain protein (MADD). This interaction leads to the activation of the ERK signaling pathway, thereby contributing to the pathogenesis of ICC (117).

#### 5.1.4 Hepatocellular carcinoma

Hepatocellular carcinoma (HCC) represents a significant global cancer burden, with an incidence rate that is increasing and closely associated with advanced liver disease (118). Lactate, a byproduct of metabolic processes, serves as a substrate for histone lactylation modifications. Research has elucidated that lactylation at lysine 9 (K9) and lysine 14 (K14) sites on histone H3, induced by lactate, facilitates the progression of HCC (119). Glypican-3 (GPC3), a protein intimately associated with tumorigenesis and tumor progression, has been identified to play a pivotal role. Experimental evidence has shown that GPC3 knockdown can diminish the levels of lactate and c-myc lactylation, thereby decreasing the stability and expression of c-myc, and retarding the deterioration of HCC (120). Furthermore, a separate study has demonstrated that cyclin E2 (CCNE2) lactylation at lysine 348 (K348) promotes HCC cell proliferation (121). Global lactylome profiling conducted on a prospectively collected hepatitis B virus-related HCC cohort has revealed that adenylate kinase 2 lactylation at lysine 28 (K28) in HCC cases markedly impairs adenylate kinase 2 activity and function, dampens apoptotic signaling, and accordingly, stimulates HCC proliferation and migration, correlating with poor patient prognosis (122). Lactate is also instrumental in stabilizing and enhancing the function of regulatory T cells. Lactate can induce lactylation at the lysine 72 (K72) site of MOESIN, thereby potentiating the signaling interface between TGF-β and its downstream SMAD3 through TGF-β receptor I, thereby amplifying Treg development within the TME (41). CENPA lactylation at lysine 124 (K124) augments its own activation and synergizes with YY1 as a transcriptional regulator to propel HCC progression (123). Liver fibrosis is a critical intermediary stage in the progression to liver cancer, and early detection and management of fibrosis, along with the mitigation or removal of carcinogenic elements, are crucial preventive strategies. It has been observed that the abundant lactate produced by activated hematopoietic stem cells can induce histone lactylation, thereby affecting hematopoietic stem cell fate. Lactylation of lysine residues at H3K9, H3K18, H4K8, and H4K12 can modulate the expression of genes involved in glucose metabolism, thereby accelerating the fibrotic process in the liver (124).

#### 5.1.5 Pancreatic ductal adenocarcinoma

Pancreatic ductal adenocarcinoma (PDAC) is the most difficult cancer to treat and has a high mortality rate (125). Severe hypoxia and hypovascular tumor microenvironment are one of its main characteristics. The Kla of nucleolar and spindle-associated protein 1 (NUSAP1) in PDAC cells inhibits the degradation of NUSAP1 protein and increases the expression of NUSAP1, thereby forming a NUSAP1-LdHA-glycolysis-lactate feedforward circuit, which contributes to the metastasis and invasion of PDAC (126). Recent studies have found that H3K18la level is elevated in PDAC and correlated with patient prognosis. H3K18la is enriched at the promoter and activates the transcription of mitotic checkpoint regulators TTK and BUB1B. The positive feedback loop of glycolysis-H3K18LA-TTK/BUB1B exacerbates PDAC dysfunction (127). Lactate-induced lactylation of NMNAT1 at lys128 enhances its nuclear localization and maintains the enzyme activity to maintain the nuclear NAD+ salvage pathway and promote the survival of pancreatic cancer cells under glucose deprivation conditions (128).

#### 5.1.6 Gastric cancer

Gastric cancer (GC) is the fifth most prevalent cancer and the third leading cause of cancer-related mortality worldwide (129). In GC, analysis of AGS cells revealed 2375 Kla sites across 1014 proteins. The findings demonstrated that the Kla level in GC is substantially elevated compared to adjacent normal tissues, and this lactylation abundance is correlative with a poorer prognosis in GC patients (130). Furthermore, the lactylation score has been identified to have a significant association with overall survival and metastatic potential in GC. Increased lactylation scores are associated with a reduced sensitivity of GC cells to immune checkpoint inhibitors (ICIs), indicating a higher propensity for immune evasion and tumor immune dysfunction (131). Recent research has uncovered that AARS1 functions as a lactate transferase that detects intracellular lactate levels and translocates to the nucleus to mediate lactylation, thereby activating the YAP-TEAD complex. AARS1 is also a Hippo target gene, creating a positive feedback loop with YAP-TEAD that enhances GC cell proliferation. Another study has shown that H3K18 lactylation upregulates VCAM1 transcription, activates the AKT-mTOR signaling pathway, and facilitates tumor proliferation and metastasis (132).

### 5.2 Respiratory cancers

#### 5.2.1 Lung cancer

Lung cancer is a multifaceted disease entity, characterized by a spectrum of histological and molecular subtypes, with non-small cell lung cancer (NSCLC) being the most prevalent. Extensive research has elucidated the role of lactate in facilitating entry into and augmenting the TCA cycle. Notably, elevated expression of LDH has been linked to adverse clinical outcomes in NSCLC patients. Experimentally, lactate-exposed NSCLC cell lines have demonstrated transcriptional up-regulation of TCA cycle enzymes, such as succinate dehydrogenase (SDH) and isocitrate dehydrogenase 3 (IDH3G), while concurrently exhibiting down-regulation of glycolytic enzymes, including hexokinase 1 (HK-1), glucose-6-phosphate dehydrogenase (G6PD), and pyruvate kinase muscle isozyme M (PKM). Chromatin immunoprecipitation (ChIP) assays have underscored the enrichment of histone lactylation at the promoters of HK-1 and IDH3G, suggesting a regulatory mechanism for lactate metabolism in NSCLC (133). Hypoxia, a defining feature of the TME, has been shown to foster glucose utilization and lactate production, thereby SOX9 lactylation and augmenting stemness, migratory, and invasive capacities of NSCLC cells by promoting glycolysis (134). Histone lactylation also plays a pivotal role in mediating tumor microenvironmental remodeling and facilitating immune evasion. In the context of NSCLC, H3K18la has been implicated in the activation of POM121 transcription, which in turn enhances the nuclear translocation of MYC and directly binds to the CD274 promoter, ultimately up-regulating PD-L1 expression and potentiating immune evasion (135). Furthermore, bioinformatics analysis has revealed a significant upregulation of BZW2 expression in lung adenocarcinoma (LUAD) tissues, which contributes to the malignant progression of LUAD by facilitating lactate production through glycolysis and promoting IDH3G lactylation (136). Regarding resistance to chemotherapy, the study identified a subpopulation of lung cancer brain metastatic cells (PC9-BrM3) that exhibited significant resistance to pemetrexed (PEM). Aldo-keto reductase family 1B10 (AKR1B10) was found to promote glycolysis and elevate the levels of histone lactylation (H4K12la) by regulating LDHA expression and lactate production. The activation of CCNB1 transcription has been shown to accelerate DNA replication and the cell cycle, thereby promoting the acquisition of PEM resistance in lung cancer brain metastases (137).

### 5.3 Urologic cancers

#### 5.3.1 Bladder cancer

Bladder cancer (BCa) represents the most frequent malignancy of the urinary tract and is among the most commonly diagnosed cancers globally (138). Investigations have revealed that elevated levels of H3K18 lactylation in human BCa are inversely associated with patient prognosis, and this modification can modulate a spectrum of malignant attributes in tumors (139). Furthermore, YBX1 and YY1, the pivotal transcription factors propelled by H3K18la, have been implicated in the cisplatin resistance observed in BCa (140).

#### 5.3.2 Renal cell carcinoma

Renal cell carcinoma (RCC) constitutes one of the three prevalent malignant tumors within the urinary system, arising from the epithelial cells of the proximal renal tubules. Research has demonstrated that the extent of histone lactylation is negatively correlated with the prognosis of patients afflicted with clear cell renal cell carcinoma (ccRCC). This modification has been shown to facilitate the progression of ccRCC by enhancing the transcription of platelet-derived growth factor receptor β (PDGFRβ). Remarkably, PDGFRβ signaling itself can induce histone lactylation levels to rise, establishing a positive feedback loop that reinforces the cancerous process throughout the carcinogenic journey of ccRCC (141).

#### 5.3.3 Prostate cancer

Prostate cancer (PCa) continues to be the most frequently diagnosed noncutaneous malignancy in males (142). Analyzes of single-cell and bulk transcriptomic datasets have implicated the hyaluronic acid (HA)-binding protein KIAA1199 in the glycolytic, hypoxic, and angiogenic pathways within PCa. The elevated lactate levels resulting from lactate uptake by PCa cells via MCT1 contribute to the lactylation and stabilization of HIF-1α. As a transcriptional activator of KIAA1199, HIF-1α can stimulate the transcription of KIAA1199 and subsequent mediation of a pro-angiogenic effect by KIAA1199 (143). Additionally, lactate treatment has been observed to enhance the expression of hypoxia-inducible factor (HIF)-1α, PD-L1, and H3K18 acetylation in PCa cells, which in turn promote tumor angiogenesis (144).

#### 5.3.4 Ocular melanoma

Ocular melanoma represents the most prevalent intraocular malignancy in adults and poses a significant diagnostic challenge (145). Prior research has uncovered a marked elevation in the level of histone lactylation (H3K18la) in ocular melanoma specimens compared to normal controls, an increase that correlates with a poorer prognosis in patients. In this context, H3K18la has been demonstrated to enhance the transcription of YTHDF2. The upregulation of YTHDF2 facilitates the recognition and degradation of m6A-modified PER1 and TP53 mRNA, thereby promoting the proliferation and migration of ocular melanoma cells (146). Recent investigations have further elucidated that histone lactylation augments ALKBH3 expression, while simultaneously attenuating the formation of promyelocytic leukemia protein (PML) condensates, a tumor suppressor, and exacerbating malignant transformation through the removal of m1A methylation in SP100A, thereby facilitating cancer progression (147).

#### 5.3.5 Acute myeloid leukemia

Acute myeloid leukemia (AML) is the most prevalent subtype of acute leukemia, with incidence rates that rise with advancing age (148). Investigations have revealed that STAT5 is significantly overexpressed in AML, a phenomenon that activates the promoters of genes associated with glycolysis and augments the glycolytic activity within AML cells. The consequent buildup of lactate triggers the nuclear translocation of E3BP, which in turn promotes histone lactylation. The elevated level of histone lactylation serves to enhance the transcription and expression of PD-L1, thereby fostering an immunosuppressive microenvironment within AML (149).

#### 5.3.6 Anaplastic thyroid cancer

Anaplastic thyroid carcinoma (ATC) is an infrequent yet lethal malignancy (150). The Warburg effect, characterized by aerobic glycolysis, is upregulated in ATC, leading to an elevation in cellular lactate levels. This increase in lactate promotes a concomitant rise in protein lactylation across the cell. Specifically, H4K12la plays a pivotal role in activating the expression of several genes crucial for ATC proliferation (151).

#### 5.3.7 Breast cancer

Breast cancer is the most prevalent cancer affecting women and the second most frequently diagnosed cancer globally (152). Research has demonstrated that AARS1 catalyzes the formation of an ATP-dependent lactate-AMP intermediate and covalently transfers a lactyl group to lysine residues, facilitating the lactylation of substrate proteins. Specifically, the AARS1-catalyzed lactylation at positions K120 and K139 within the DNA-Binding Domain (DBD) of p53 leads to its inactivation, resulting in compromised DNA binding, diminished liquid-liquid phase separation, and decreased transcriptional activity, all of which contribute to tumorigenesis (91). Potassium Two-Pore Channel Subfamily K Member 1 (KCNK1) is upregulated in various cancers and is associated with a poor prognosis in breast cancer patients. KCNK1 enhances glycolysis and lactate production in breast cancer cells by binding to and activating LDHA, thereby promoting histone Kla. This ultimately facilitates the proliferation, invasion, and metastasis of breast cancer (153).

#### 5.3.8 Glioblastoma

Glioblastoma (GBM) is the most prevalent and aggressive form of primary brain tumor in adults (154). Prior research has demonstrated that the NF-κB signaling pathway facilitates the Warburg effect in GBM, resulting in elevated lactate levels that lead to lactylation at H3K18. This lactylation augments the transcriptional activity of the downstream gene LINC01127, thereby enhancing the self-renewal and detrimental cancer progression of GBM cells through the MAP4K4/JNK/NF-κB axis (155). Hypoxic conditions promote lactate accumulation in glioma cells via glycolysis, which is then taken up by macrophages, triggering M2 polarization via the MCT1/H3K18la/TNFSF9 axis, thereby significantly exacerbating the malignancy of glioma cells (156). In a mouse model of GBM, it has been observed that histone lactylation can curtail T cell activity. Lactate intracellularly upregulates the expression of CD39 and CD73 through H3K18 lactylation, resulting in the depletion of CD8 T cells and the proliferation of Tregs (157). Tumor-associated macrophages (TAMs), consisting primarily of resident microglia (MGs) and monocyte-derived macrophages (MDMs), are intimately linked to the immunosuppressive tumor microenvironment. Monocyte-derived macrophages are predominantly responsible for the immunosuppressive response in GBM. Activation of the PERK pathway augments GLUT1 expression, bolstering glycolysis and macrophage metabolism, which in turn increases IL-10 expression via lactylation, thereby mediating the immunosuppressive response in MDMs (158). Vasculogenic mimicry (VM) provides a blood supply to tumor cells independent of endothelial cells. Tumor-specific molecular markers for VM include VEGFR2 and VE-cadherin. The MAPK6p4-encoded functional peptide P4-135aa phosphorylates KLF15 at S238, thereby enhancing KLF15 protein stability and nuclear translocation. As a transcriptional activator, KLF15 binds to and promotes VEGFR2 and the lactylation of VE-cadherin, fostering the development of GBM VM (159).

#### 5.3.9 Endometrial carcinoma

Endometrial cancer (EC) has emerged as a leading gynecological malignancy in developed countries worldwide, characterized by a noted escalation in its incidence rates (160, 161). Extensive research has revealed that histone lactylation enhances the aggressive biological behavior of EC cells by stimulating the PI3K/AKT/HIF-1α signaling pathway, which is facilitated through the upregulation of USP39 expression (162).

#### 5.3.10 Cervical cancer

Cervical cancer ranks as the fourth most prevalent cancer among women globally (163). Recent investigations have demonstrated that lactate can induce lactylation at lysine 72 of Discoidin, CUB, and LCCL domain-containing type I (DCBLD1), thereby stabilizing DCBLD1 expression. DCBLD1, in turn, advances the progression of cervical cancer by increasing the expression and activity of G6PD, impeding its autophagic degradation, and activating the pentose phosphate pathway (PPP) (164). Human papillomavirus type 16 (HPV16), a high-risk strain, is the most frequently identified etiological agent in cervical cancer cases. The persistent expression of the E6 and E7 oncogenes is a fundamental driver of cervical carcinogenesis. Recently, however, it has been discovered that HPV16 E6 facilitates the formation of G6PD dimers by preventing the lactylation of G6PD. This event serves to upregulate the enzymatic activity of G6PD, thereby activating the pentose phosphate pathway (PPP), which, in turn, fosters the proliferation of cervical cancer cells (165).

#### 5.3.11 Ovarian cancer

Ovarian cancer(OC) currently stands as the fifth leading cause of cancer-related mortality in women within the United States, and it annually claims the lives of approximately 140,000 women globally (166). Emerging research has revealed that lactate stimulates the expression of CCL18 by inducing lactylation at H3K18 in macrophages, thereby facilitating the tumorigenesis of OC (167).

Lactylation plays a multifaceted role in cancer pathobiology, encompassing five primary functions (1): the augmentation of drug resistance, (2) the mediation of immunosuppression, (3) the promotion of invasion, (4) the enhancement of proliferation, and (5) the facilitation of angiogenesis. The mechanisms by which lactylation exerts these effects in cancer include (1) the upregulation of oncogene expression through the enhancement of histone lactylation and (2) the modulation of non-histone protein stability and activity through the enhancement of non-histone lactylation.

## 6 Lactylation as diagnostic biomarkers

Lactate is renowned within the scientific community for its role as an energy source and metabolic by-product, and its derivative, lactylation, has revolutionized its significance in disease pathogenesis. Extensive research has established lactylation as a diagnostic biomarker for tumor progression and metastasis (**Table 1**). The Lactylation-Related Gene Set encompasses a collection of genes that are either directly involved in the lactylation process or related to it, spanning the full spectrum of this modification’s biological aspects. This includes enzymes responsible for adding and removing lactyl groups, as well as genes whose expression is influenced by this epigenetic mark. These genes are identified through experimental research or bioinformatic analysis and are associated with lactylation either directly or indirectly (168). In the context of tumors, the Lactylation-Related Gene Set often mirrors the extent of lactylation, which is indicated by the expression levels of lactyltransferases, gene expression profiles, and lactate concentrations. This is primarily mediated through its impact on tumor cell metabolism and gene expression. To validate and enhance the relevance of the Lactylation-Related Gene Set, further analysis is necessary, including bioinformatics assessment, functional enrichment studies, and examination of clinical correlations. This comprehensive approach aims to yield novel insights and potential biomarkers that could contribute to the diagnosis, treatment, and prognosis assessment of cancer (131, 169–173). Notably, Analysis of databases revealed that 13 genes are associated with hypoxia-glycolysis-lactylation (HGLRG) signature, and an HGLRG risk score identified GC patients with a better prognosis in the low-risk category. HGLRG signature significantly impact GC survival predictions and may provide a valuable clinical model for gastric cancer (174). Furthermore, researchers have proposed that the lactylation score can serve as a prognostic indicator for GC malignancy progression and immune evasion, potentially guiding therapeutic responses to ICIs in GC (131). Differentially expressed lactylation-related genes (LRGs) between multiple myeloma (MM) and normal samples were identified from the Gene Expression Omnibus database, highlighting differences in biological pathways, immune status, and chemotherapy drug sensitivity between high-risk and low-risk MM patients, suggesting LRGs as a promising biomarker for early high-risk MM detection and prognosis prediction (175). In colorectal cancer (CRC), a 23-gene Lactylation-Related Gene risk model has been developed to forecast patient prognosis, potentially holding a pivotal role in refining CRC management and treatment outcomes (169). Analysis of the Cancer Genome Atlas (TCGA) database yielded 16 prognostic differentially expressed LRGs, enabling the construction of a lactylation-related gene signature, with high-risk score patients experiencing poor outcomes and low-risk score patients responding more favorably to targeted therapies and immunotherapy, offering potential clinical utility as a biomarker for HCC treatment efficacy (176). Additionally, risk models centered on HDAC1 and HDAC2 have been shown to forecast the prognosis of HCC patients (177). PCa patients in the high-risk group exhibit a higher prevalence of regulatory T cells and M2 macrophages, increased tumor mutation burden, and a correspondingly poorer prognosis, rendering the LRGs prognostic model an effective tool for predicting DFS and treatment response in PCa (178). In PDAC, 10 differentially expressed LRGs predictive of prognosis were identified, leading to the construction of a lactylation-related signature incorporating five OS-related genes (SLC16A1, HLA-DRB1, KCNN4, KIF23, and HPDL), capable of forecasting OS, immune status, and treatment response in pancreatic cancer patients (179). Osteosarcoma (OS) research utilizing the TARGET database identified 329 LRGs, pinpointing 27 crucial genes, among which MYC and GOT2 were pivotal, suggesting considerable potential for the prognosis and treatment of OS using the established risk model (180). LRGs significantly influence breast cancer by modulating tumor growth, the immune microenvironment, and drug responses, positioning them as promising prognostic markers and therapeutic targets (170). Single-cell RNA sequencing (scRNA-seq) data analysis has identified LRGs that contribute to the establishment of a prognostic signature, with high score patients demonstrating poor overall survival (OS) and a better response to immunotherapy, potentially enhancing the survival prediction and immunotherapy response in gliomas (181). Studies have also shown that LRGs are involved in tumor classification and immunity in OC patients, with the lactylation-related gene signature effectively differentiating and predicting OC prognosis, thereby offering a valuable tool for OC management (182).

**Table 1:** Lactylation as diagnostic biomarkers in cancers.

Lactylation-related genes	Source Databases	Disease	Result	References
CP, BGN, DUSP1, SERPINE1, CLDN9, PAK2, TP53, HK3, PAXIP1, NUP50, EFNA3, ESRRB, OGT	TCGA GEO	Gastric Cancer	It is effective in predicting survival and providing a practical clinical model for gastric cancer.	(174)
EFNA3, VCAN, PLOD2, HBB, NUP50, STC1	TCGA GEO	Gastric cancer	It can guide the therapeutic response to immune checkpoint inhibitors against GC and is also expected to be a potential therapeutic target for GC and diagnostic markers.	(131)
TRIM28, PPIA, SOD1, RRP1B, IARS2, RB1, PFN1, PRCC, FABP5	GEO	Multiple myeloma	It is a promising biomarker for MM that can effectively early distinguish high-risk patients and predict prognosis.	(175)
RBM17, TERF2IP, AP2M1, NR1H2, MED10, HSPA1B, ARL4C, LY6E, ARPC1B, HSPB1, SLC2A3, RBM3, LGALS4, PTTG1, H2AFY, HMGN2, LEPROTL1, LITAF, ACTG1, RAB5C, METTL9, UBE2I, SFPQ	TCGA GEO	Colorectal cancer	It has significant potential to improve the management of patients with colorectal cancer and enhance treatment outcomes	(169)
ARID3A/DRIL1, CCNA2, DDX39A/DDX39, EHMT2/KMT1C, FABP5, G6PD, H2AX/H2AFX, HMGA1, KIF2C/KNSL6, MKI67, PFKP, PKM2, RACGAP1/CYK4, RFC4, STMN1, TKT	TCGA ICGC	Hepatocellular carcinoma	It can be used as a biomarker for effective clinical treatment of HCC	(176)
EP300, HDAC1-3		Hepatocellular carcinoma	It can be used to predict the prognosis of HCC patients	(177)
ALDOA, DDX39A, H2AX, KIF2C, RACGAP1	TCGA	Prostate cancer	It can effectively predict the disease-free survival and therapeutic responses of PCa patients	(178)
SLC16A1, HLA-DRB1, KCNN4, KIF23, HPDL	GTEx TCGA GEO	pancreatic adenocarcinoma	It can predict overall survival, immune status, and treatment response in patients with pancreatic adenocarcinoma	(179)
MYC, GOT2	TARGET	Osteosarcoma	It has great potential in the treatment of osteosarcoma	(180)
ARID3A, CCNA2, DDX39A, EHMT2, FABP5, G6PD, H2AX, HMGA1, KIF2C, MKI67, PFKP, PKM2, RACGAP1, RFC4, STMN1, TKT, EFNA3, VCAN, PLOD2, HBB, NUP50, STC1	TCGA GSE20685	Breast cancer	It can be used as a prognostic marker and a potential therapeutic target for breast cancer	(170)
LDHA, LDHB, LDHC, LDHD, PDHA1, PDHB, PDHX, PDHE1, PKM, PKLR, ENO1, ENO2, ENO3, PFKP, PFKM, TPI1, GAPDH, MCT1, MCT2, MCT3, MCT4, MCT5, SLC16A7, SLC16A9, SLC16A11 ALDH1, ALDH2, ALDH3, ALDH4 ALDH5, ALDH6, ALDH7, ALDH9, ALDH16	Tumor Immune Single-Cell Hub, Molecular Signatures	Glioma	It can be used for robust survival prediction and immunotherapeutic response	(181)
SNRPA1, MPHOSPH6, POLDIP3, RB1, AHNAK, MAGOHB, CALM1, EP300, HDAC1, HDAC2, HDAC3, SIRT1, SIRT2, SIRT3	TCGA	Ovarian cancer	It can effectively distinguish and predict the prognosis	(182)

## 7 Targeting lactate and lactylation for cancers therapy

In the intricate tapestry of cellular carcinogenesis, lactate plays a multifaceted role, encompassing metabolic reprogramming, alterations in the TME, signal transduction, and immune suppression. Extensive research has elucidated that lactate possesses the capability to modulate the trajectory of cancer progression. The build-up of lactate within tumor cells facilitates immune evasion and exerts an inhibitory influence on immune-mediated cell death (19, 29, 183–185). Conversely, the reduction of lactate levels has been demonstrated to curtail tumor growth, offering novel avenues for cancer therapeutic strategies.

The recent unveiling of lactylation, a PTM involving the lactate attachment to proteins, has generated significant excitement across the scientific community. This novel mechanism has garnered attention in diverse fields and has emerged as a pivotal player in cancer research. Clinically, the advent of lactylation opens up fresh prospects for the diagnosis and management of cancer patients, holding the potential to revolutionize cancer therapies.

### 7.1 MCTs

MCTs, which belong to the solute carrier transporter family, are pivotal in maintaining intracellular and extracellular lactate homeostasis. The transport capabilities of MCT1 and MCT4 are crucial for the regulation of lactate exchange in and out of cells, a process that is substrate-concentration dependent (186–188). Moreover, these transporters are involved in the shuttling of various carboxylates, including pyruvate, β-hydroxybutyrate, and acetate (189, 190). A range of MCT inhibitors exists, with syrosingopine, AR-C155858, 7ACC2, BAY8002, SR13800, and AZD3965 demonstrating inhibitory potential against MCTs. To date, AZD3965 is the only inhibitor to have progressed to clinical trials (NCT 01791595) (191). The elevated lactate levels within the TME are facilitated by MCTs for delivery to tumor cells or immune cells, thereby influencing tumor behaviors such as immune evasion, proliferation, and invasion. AZD3956, an MCT1 inhibitor, has been shown to alter cellular metabolism and enhance the infiltration of anti-tumor immune cells, impeding tumor growth in murine models (192). Extensive research indicates that AZD3956, which inhibits MCT1, can curtail the proliferation of various tumors (193–195), including lung cancer (196), breast cancer (197, 198), lymphoma (199–201), and colon cancer (200). Conversely, MCT4 inhibition results in lactate accumulation within cancer cells, leading to cell death (202). Notably, MCT4 inhibition has been found to augment the therapeutic efficacy of HCC treatment with anti-PD-1 therapy by bolstering T-cell infiltration and immune response (187). Additionally, CD147, a chaperone essential for the proper functioning of both MCT1 and MCT4, significantly contributes to tumor migration, invasion, and metastasis. Targeting CD147 to suppress the activity of MCT1 and MCT4 holds promise for cancer therapy, offering novel insights into treatment strategies (203).

### 7.2 LDHs

Lactate dehydrogenases (LDHs) is an enzyme ubiquitously expressed within cellular systems, encompassing five primary isoenzymes. In the clinical diagnostic context, LDH A and LDH B are the primary isoforms of interest. Notably, studies have elucidated that LDH A is overexpressed in a variety of malignant tissues and is correlative with a diminished patient survival prognosis. The modulation of LDH activity emerges as a potential strategy to augment T-cell infiltration and potentiates the efficacy of immunotherapy, such as ICIs. Utilizing a humanized mouse model for NSCLC, the inhibition of LDH with agents such as oxalate and the monoclonal antibody pembrolizumab has demonstrated a significant capacity to retard tumor progression, with a synergistic effect observed upon combination (204). This phenomenon has been recapitulated in HCC, where the conjunction of anti-PD-1 therapy with LDH inhibitors has revealed a superior anti-tumor response compared to monotherapy (41). Furthermore, the blockade of LDH A, by competitive inhibition, is established to exert anti-leukemic effects in T-cell acute lymphoblastic leukemia. Oxalate effectively curtails cell proliferation and induces apoptosis in Jurkat and DU528 cell lines (205). Moreover, the specific silencing of LDH A through small interfering RNA (siRNA) has been shown to induce oxidative stress and cellular demise, thereby impeding carcinogenic progression (206). In LDH A deficient tumors treated with anti-PD-1 therapy, a reduction in regulatory T cell infiltration was observed, suggesting that the manipulation of LDHA within the TME can substantially enhance the efficacy of anti-PD-1 treatment (206). Concomitantly, the compound 1-(phenylseleno)-4-(trifluoromethyl) benzene (PSTMB) has been identified to inhibit LDH A activity, thereby curbing tumor growth, primarily through the induction of apoptotic cell death (207).

### 7.3 HATs

Histone acetyltransferases (HATs) serve as pivotal transcriptional regulators, principally by appending acetyl groups to the N-termini of histones, which in turn modulates the architecture and functional integrity of chromatin. Additionally, HATs are capable of introducing lactyl groups onto lysine residues. Given the intricate association between lactate metabolism and the progressive growth and proliferation of various malignancies, HATs have emerged as prime therapeutic targets for the treatment of cancerous disorders. Garcinol, an extractive natural compound derived from the botanical genus Garcinia, has been identified as a robust inhibitor that specifically targets the P300 and PCAF protein domains. Treatment with garcinol leads to a dose-dependent decrement in the nuclear protein levels of P300/CBP and phosphorylated Smad2/3, accordingly inhibiting the metastatic potential of esophageal cancer cells (208). Experimental evidence has also demonstrated that the administration of C646, a P300-specific inhibitor, can augment the cellular response to gemcitabine, prompting apoptotic cell death and thereby potentiating the cytotoxic effects of gemcitabine on pancreatic cancer cells (209). Andrographolide (Andro), utilized in the realm of cancer therapeutics, has been observed to curtail breast cancer progression by suppressing COX-2 expression and sequestering the P300 signaling cascade, in conjunction with thwarting VEGF-mediated angiogenesis (210).

In conclusion, the strategic targeting of critical enzymes within the glycolytic pathway, lactate transporters, or enzymes responsible for lactylation, has garnered substantial foundational research in the realm of antitumor therapy. Such interventions hold the potential to markedly enhance the efficacy of tumor treatment regimens, and when coupled with immune checkpoint inhibitors, a synergistic therapeutic response can be observed. Consequently, the manipulation of lactate generation and transport, the modulation of lactylation, and the regulation of the TCA cycle emerge as promising avenues for precision tumor therapy. Nonetheless, the clinical deployment of inhibitors aimed at curbing tumor metastasis remains in its infancy, and the translation of these inhibitors into clinical practice is fraught with challenges, necessitating extensive preclinical and clinical research to establish their safety and therapeutic efficacy.

## 8 Conclusions and future perspectives

As a metabolic by-product of the glycolytic pathway, the accumulation of lactate within the TMA has long been recognized as a hallmark of cancer. The Warburg effect has elucidated various functions of lactate, encompassing the regulation of energy metabolism, cell signaling, immunosuppression, angiogenesis, invasion, and metastasis, thereby influencing a multitude of cellular phenotypes-positioning lactate as a pivotal player in cancer biology. In 2019, Kla was identified as a novel protein PTM that impacts the metabolic reprogramming of cancer. Serving as a nexus between metabolism and epigenetics, Kla can up-regulate the expression of genes associated with metabolism via diverse mechanisms, thereby fostering tumor growth, metastasis, and invasion. However, the investigation into the epigenetic regulation of lactylation remains in its nascent stages, with numerous scientific questions yet to be answered. The endeavors to decipher the mechanisms underlying histone and nonhistone lactylation modifications in tumor initiation and progression are fraught with challenges, rendering lactylation a potential and promising therapeutic target in the fight against cancer.

In the domain of oncology, research on Kla represents an emerging field aimed at yielding deeper insights and understanding of cancer biology. Here, we comprehensively review the role and underlying mechanisms of lactylation in the initiation and progression of various malignancies, as well as its potential as a therapeutic target. Studies indicate that lactylation is intricately linked to the deterioration of cancer and can facilitate tumorigenesis and metastatic spread. Given that research into lactylation in cancer is still in its relative infancy, with the specific therapeutic mechanisms yet to be fully elucidated, significant challenges remain in harnessing its therapeutic potential and application. Nonetheless, this field offers novel concepts and directions for future basic research and clinical translation. Investigations have revealed that the level of lactylation can serve as a biomarker for the diagnosis and prognostic assessment of cancer, aiding in the formulation of personalized clinical treatment strategies and the tailoring of therapies based on individual lactylation levels, which may enhance targeted treatment efficacy and broaden the therapeutic benefit. Further exploration of the lactylation mechanism within the TME, the discovery of novel regulatory targets, the development of therapeutics targeting lactylation-related enzymes and proteins, the integration of these approaches with conventional treatment modalities, and the enhancement of immunotherapeutic strategies are promising avenues to improve cancer treatment outcomes, offering new hope and expanding the array of treatment options for patients with cancer.
